# A Statistical Definition of Epidemic Waves

**DOI:** 10.3390/epidemiologia4030027

**Published:** 2023-07-03

**Authors:** Levente Kriston

**Affiliations:** Department of Medical Psychology, University Medical Center Hamburg-Eppendorf, Martinistr. 52, D-20246 Hamburg, Germany; l.kriston@uke.de

**Keywords:** COVID-19, SARS-CoV-2, epidemiologic methods, Bayesian analysis, computational biology

## Abstract

The timely identification of expected surges of cases during infectious disease epidemics is essential for allocating resources and preparing interventions. Failing to detect critical phases in time may lead to delayed implementation of interventions and have serious consequences. This study describes a simple way to evaluate whether an epidemic wave is likely to be present based solely on daily new case count data. The proposed measure compares two models that assume exponential or linear dynamics, respectively. The most important assumption of this approach is that epidemic waves are characterized rather by exponential than linear growth in the daily number of new cases. Technically, the coefficient of determination of two regression analyses is used to approximate a Bayes factor, which quantifies the support for the exponential over the linear model and can be used for epidemic wave detection. The trajectory of the coronavirus epidemic in three countries is analyzed and discussed for illustration. The proposed measure detects epidemic waves at an early stage, which are otherwise visible only by inspecting the development of case count data retrospectively. Major limitations include missing evidence on generalizability and performance compared to other methods. Nevertheless, the outlined approach may inform public health decision-making and serve as a starting point for scientific discussions on epidemic waves.

## 1. Introduction

“Surveillance is the collection, collation, and analysis of data and the dissemination to those who need to know so that an action can result” [1]. Surveillance is an essential component of detecting and controlling infectious disease epidemics because it enables the timely allocation of resources and the initiation of interventions as soon as they are needed. In addition, by providing forecasts, they try to prepare the healthcare system and the public for future events. Commonly, surveillance, monitoring, and forecasting of the course of infectious disease epidemics rely heavily on epidemiological modeling [2]. Such models have attracted much attention during the severe acute respiratory syndrome coronavirus 2 (SARS-CoV-2) pandemic [3,4,5].

Epidemiological models of infectious disease transmission are usually categorized as being primarily theory-driven (‘mechanistic’) or data-driven (‘phenomenological’) [2,3]. Theory-driven models are based on concepts from established scientific disciplines (e.g., biology, virology, infectiology). These models use knowledge about the mechanisms that drive transmission dynamics to compare different possible scenarios of the development of epidemics. Data-driven models, on the other hand, intend to find a mathematical function that fits observed data. By doing so, they are ignorant of domain knowledge regarding transmission processes and only aim to minimize the discrepancy between observed and model-implied data.

The course of infectious disease epidemics is frequently described by referring to ‘waves’. Waves can easily be imagined and displayed visually, and thus they can support comprehensible communication between policymakers, scientists, and the public. However, a consensual definition of what constitutes an epidemic wave is currently missing [6,7,8]. Some consider the term a useful metaphor referring to a sustained upsurge (frequently called ‘spike’) in the number of sick individuals (cases) [9]. From an even broader perspective, a complete wave includes a rise in the number of cases, a defined peak, and a decline. Although this approximate definition is helpful, it is not sufficiently exact for scientific inquiry. It leaves considerable room for subjectivity. For example, we do not know how strong a ‘rise’ in the number of cases has to be and how long it has to be ‘sustained’ to be certain enough to talk about a ‘wave’.

Modeling epidemic waves cannot be clearly positioned on the theory-driven vs. data-driven continuum of epidemiological approaches. Although it attempts to quantify the defined concept of a ‘wave’, the definition is usually blurry, and the suggestions for quantification are rather descriptive than predictive. At the same time, the aim of identifying an epidemic wave is primarily motivated by the need for some kind of prognostic, or at least diagnostic, information about the state of the epidemic at a given time point. The unclear role of the concept of a ‘wave’ in traditional epidemiological models may be one of the reasons why quantitative research on this topic is scarce.

The present work is focused only on the first rising phase of epidemic waves. Autoregressive integrated moving average (ARIMA) models are frequently used for detecting possible disease outbreaks and/or epidemic waves. However, their usefulness consists “not so much in detecting outbreaks or providing probability statements, but in giving decision-makers a clearer idea of the variability to be expected among future observations” [10]. Thus, ARIMA models may play an important role in avoiding false alarms but, at the same time, may be prone to false negative findings. In addition, they do not provide a clear quantitative measure of the risk of negative development.

Recently, it has been suggested to use the mean of the effective reproduction number R (which refers to the average number of individuals infected by a single infectious individual during a running epidemic) over the past 14 days to operationalize epidemic waves [8]. This working definition is certainly useful to put discussions on epidemic waves on a more objective footing. However, as the authors acknowledge, it describes rather a ‘sustained upward period’ than an upsurge in the number of cases. In addition, by calculating the average of equally weighted data points in a defined period, it discards the temporal information that is present in the data.

Technically, the unrestricted spread of infectious diseases is commonly characterized by the exponential growth of the number of confirmed cases, while reduced virus transmission and reproduction decelerate growth to a subexponential rate [11,12]. The aim of this study was to provide a statistical measure of epidemic waves by determining whether the dynamics of an epidemic within a certain time horizon are more likely to be exponential than linear.

## 2. Materials and Methods

The proposed measure is based on the time series of the observed daily new cases, as using cumulative data can lead to biased conclusions [13]. While an exponential growth of the total case counts analytically implies an exponential growth of the daily new case counts, it is assumed here that a typical subexponential growth of the total case counts can be well approximated by a linear growth of the daily new case counts. It should be noted that this assumption is not supported by any of the mechanistic models of disease transmission known by the author. However, it is important to have a comparator model that allows for a rising number of cases in order to be able to differentiate a surge from a simple increase [8]. A linear model seems to be an optimal comparator due to its simplicity, interpretability, and wide use in modeling temporal trends.

Although the term ‘growth’ is used here, the suggested indicator does not differentiate between increasing and decreasing new case counts per se. Thus, it can detect not only exponential surges but also exponential declines. However, as the present study focused on epidemic surges, i.e., the increasing phase of epidemic waves, the proposed measure was calculated only if the exponent of the exponential function exceeded one (i.e., if the number of daily cases was increasing rather than declining).

The proposed epidemic wave indicator is a Bayes factor that quantifies the strength of evidence in support of the hypothesis that the dynamics of an epidemic are exponential rather than linear. It is calculated using the Bayesian information criterion approximation method from the coefficient of determination of two linear models [14]. The exponential model is calculated by regressing the logarithmic daily new case counts on time in a linear model. This method makes use of the fact that the linear association of a predictor with a logarithmically transformed criterion is analytically equivalent to an exponential association of the predictor and the untransformed criterion. The linear model is calculated by regressing the raw daily new case counts on time.

For the calculations, it is necessary to define a time horizon (*n* days). The wave indicator at any time point describes the development of the epidemic in the last *n* days up to the time point of calculation and should be interpreted as referring to that time interval. In the present study, the wave indicator was calculated if more than 70 percent of the daily new case numbers in the given time horizon were positive.

According to Raftery [14], the number of data points (*n*), the coefficient of determination (*R*^2^), and the number of predictors without the intercept (*p*) can be used to approximate the Bayesian information criterion (*BIC*) for linear models as

(1) $$BIC=n\times \log\left(1-R^{2}\right)+p\times \log n,$$

with log referring to the natural logarithm.

Following Wagenmakers [15], the Bayes factor (*BF*) for the support of one model over another can be calculated from the *BIC* of the two models as

(2) $$BF=\exp\left(\frac{BIC_{lin}-BIC_{exp}}{2}\right),$$

where $BIC_{lin}$ and $BIC_{exp}$ refer to the coefficient of determination in the linear and exponential models, respectively.

Merging Equations (1) and (2) leads to the formula

(3) $$BF={\left(\frac{1-R_{lin}^{2}}{1-R_{exp}^{2}}\right)}^{\frac{n}{2}},$$

where $R_{lin}^{2}$ and $R_{exp}^{2}$ refer to the coefficient of determination in the linear and exponential models, respectively.

The resulting Bayes factor expresses the strength of support for the exponential over the linear model. A value of one indicates that exponential and linear dynamics have the same probability. Values above one support exponential dynamics, while values below one support linear dynamics. If necessary, thresholds for interpretation are available, classifying a Bayes factor between 1 and 3 as weak, between 3 and 20 as positive, between 20 and 150 as strong, and above 150 as very strong evidence [14,15]. These thresholds correspond to a 75, 95, and 99 percent probability that the exponential model is true if we assume that they were equally probable before seeing the data [15]. In the present study, a 95 percent bootstrap interval was created for the Bayes factor estimates with 500 samples in order to gain an impression of uncertainty related to the data.

All analyses were performed in R version 4.1.3 [16]. The annotated code can be found in Supplementary Materials (Text S1).

## 3. Results

For illustration, the proposed Bayes-factor-based epidemic wave indicator was calculated for the coronavirus epidemic in the United States, the United Kingdom, and Germany with a time horizon of one, two, and three months, using data from the World Health Organization from initiation until 28 February 2022 [17].

The wave indicator can be visualized in a line plot with the date on the x-axis and the wave indicator for each day on which the exponent of the exponential function exceeded one (i.e., on which the number of daily cases was increasing rather than declining) on the y-axis. Shaded areas indicate bootstrap intervals to reflect uncertainty in the wave indicator estimate. The dotted, dashed, and solid horizontal lines show thresholds for positive, strong, and very strong evidence, respectively. A wave indicator above the solid line indicates very strong evidence for the presence of an epidemic wave. In the following, wave indicators are presented for the time horizon of one, two, and three months, respectively, reflecting the length of the retrospective time span used for including data in the calculation. 

**Figure 1:**
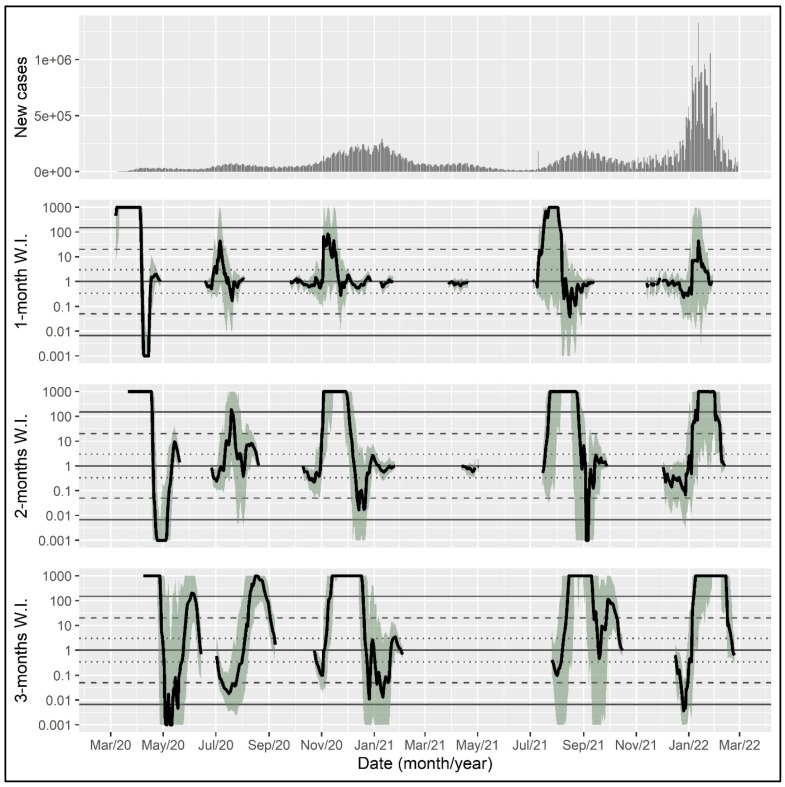
Daily new case counts and epidemic wave indicator with different time horizons in the coronavirus epidemic in the United States. Shaded areas indicate bootstrap intervals calculated with 500 samples. The dotted, dashed, and solid horizontal lines show thresholds for positive, strong, and very strong evidence, respectively. W.I., wave indicator.

Relying on daily case counts, five epidemic waves can be identified in the United States until the end of February 2022 (Figure 1). All versions of the indicator identify the first wave in March 2020 clearly. The second wave in the summer of 2020 is identified only by the 3-month version, with a substantial delay in August 2020. The third wave hitting in winter 2020/2021 has been clearly signaled by the 2- and 3-month versions already in November 2020. The fourth wave, which lasted through the late summer and autumn of 2021, has been recognized by all versions of the indicator around the beginning of August 2021, albeit these signals have substantial data-related uncertainty. The fifth wave of winter 2021/2022 has been signaled by the 3- and (with a somewhat higher uncertainty) the 2-month versions around the turn of the year.

**Figure 2:**
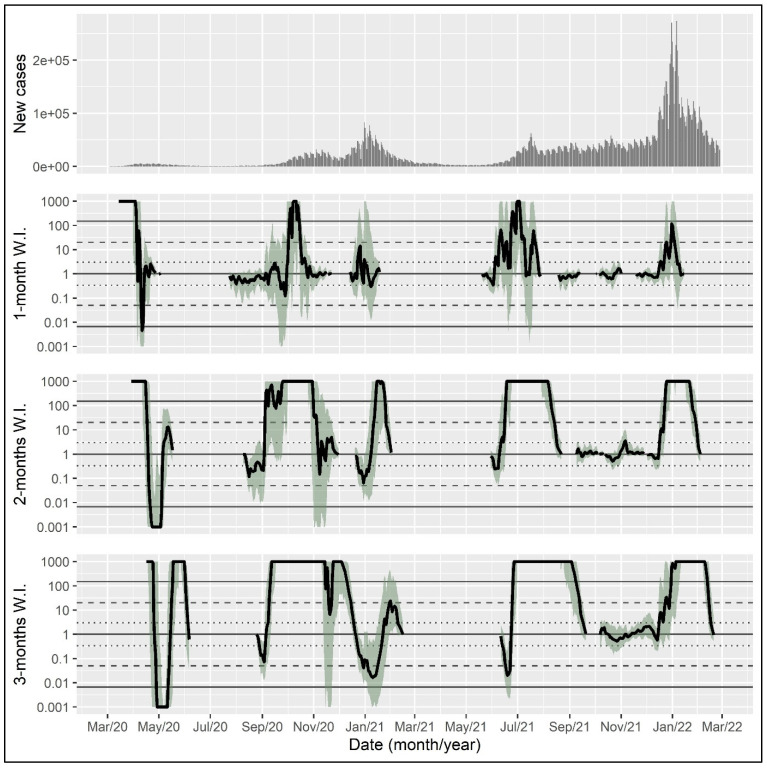
Daily new case counts and epidemic wave indicator with different time horizons in the coronavirus epidemic in the United Kingdom. Shaded areas indicate bootstrap intervals calculated with 500 samples. The dotted, dashed, and solid horizontal lines show thresholds for positive, strong, and very strong evidence, respectively. W.I., wave indicator.

Data on the daily new case counts suggest five epidemic waves in the United Kingdom up to February 2022 (Figure 2). The first surge in daily new cases in the spring of 2020 was clearly identified as a wave irrespective of the time horizon used. The second wave in the late summer of 2020 had somewhat weaker support by the indicator. The winter wave in 2020/2021 is clearly signaled by the 2- and the 3-month versions of the indicator already at the beginning of November 2020. The fourth wave in the late summer and autumn of 2021 is unequivocally recognized by all versions, with very strong evidence in July–August. The fifth wave hitting in the winter of 2021/2022 is marked by very strong evidence by the 2- and 3-month versions at the beginning of January 2022.

**Figure 3:**
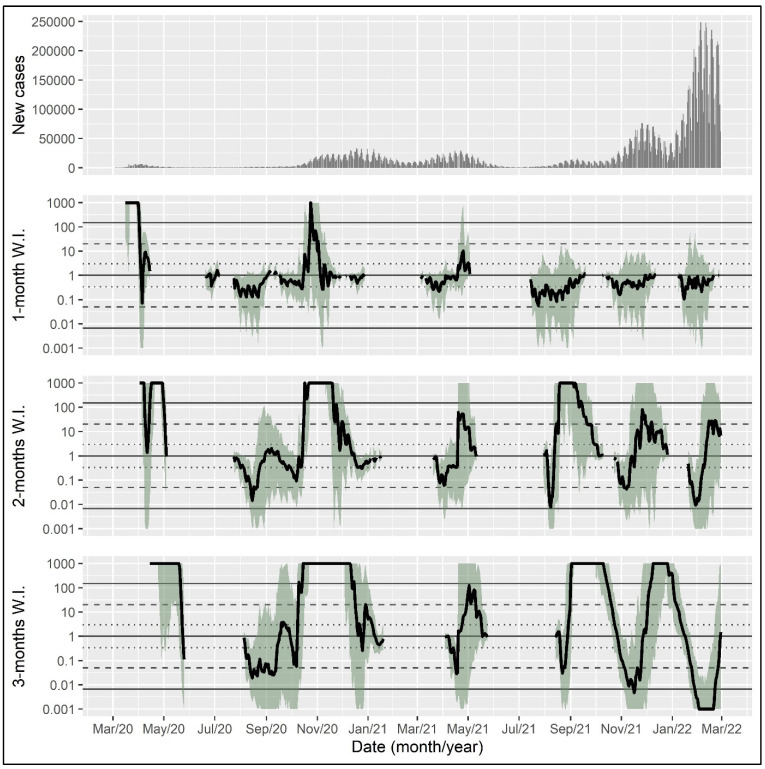
Daily new case counts and epidemic wave indicator with different time horizons in the coronavirus epidemic in Germany. Shaded areas indicate bootstrap intervals calculated with 500 samples. The dotted, dashed, and solid horizontal lines show thresholds for positive, strong, and very strong evidence, respectively. W.I., wave indicator.

The inspection of the time series of the daily new cases in Germany indicates six epidemic waves up to the end of February 2022 (Figure 3). All versions of the indicator show very strong evidence of an epidemic wave in March 2020 (first wave). Very strong evidence of a second wave is provided for the autumn of 2020, signaled by all versions around the middle of October 2020. A third wave that is apparent in the daily new cases data in the spring of 2021 is identified only weakly and with considerable uncertainty. A clear signal for a fourth wave is provided by the 2- and 3-month versions of the indicator in the late summer of 2021, even though the number of new cases is relatively low compared to the other waves. A fifth wave at the end of 2021 is clearly signaled only by the 3-month version, while the intensive sixth wave at the beginning of 2022 is not recognized by any version at the time point of the investigation.

Identifying epidemic waves from the time series of daily new case counts is challenging, even retrospectively. This is particularly true if the apparent waves follow each other swiftly and/or build upon each other. Instead of five to six waves as described above, data from all three countries are consistent with the interpretation of three ‘big’ waves, the first ending in the spring of 2020, the second running through the autumn and winter of 2020/2021, and the third centering on the winter of 2021/2022. These three ‘big’ waves are all identified very clearly and early by the proposed indicator.

## 4. Discussion

The proposed approach makes clear that judgments on epidemic waves depend on the timeframe of reference and that apparently visible patterns in case count data may provide a subjective and/or incomplete picture. The measure outlined in this study is scalable to any geographic region and takes possible irregularities of the data into account.

A central limitation of the presented approach is that it relies on the number of reported cases, which can be subject to inconsistencies due to variations in reporting and testing strategies or changing population behavior. These factors may influence the daily case counts and therefore impact the accuracy of the proposed epidemic wave indicator. Thus, the identified waves do not necessarily reflect changes in the true number of infections. Future studies may consider alternative data sources and more complex models to account for potential biases in the reported data.

However, it is unlikely that testing and reporting strategies alone are able to produce epidemic waves with very strong support from the proposed epidemic wave indicator. For example, a rapid absolute change in the number of cases due to changes in the counting or reporting of cases would also be captured by the comparator linear model. Consequently, it would not be enough to substantially increase the proposed wave indicator. Nevertheless, incremental changes and slower shifts may bias the accuracy of the suggested method. The calculation of bootstrap intervals (which should be interpreted as reference intervals rather than traditional confidence limits) can be helpful for assessing the data-related uncertainty of the calculations. This issue clearly deserves further exploration.

Another challenge is posed by the question of which time horizon should be used to calculate the wave indicator. In the examples, indicators with a longer time horizon (two and three months) seem to have worked better and more clearly for detecting epidemic waves. However, the choice is likely to depend on the characteristics of the waves of which description the measure is intended to use. For epidemics with an annual periodicity of major waves, a time horizon of several months might be appropriate. However, until clearer guidance is available, it seems reasonable to use multiple timeframes, like it was conducted in the present study. In case the findings from using different time horizons are inconsistent, further inquiry is necessary and premature conclusions should be avoided.

Another important limitation of the present study is that it did not evaluate the predictive accuracy of the proposed wave indicator. Empirical investigations of the forecasting value of epidemiological models are surprisingly scarce and consented methodological guidance is missing on how to perform such studies [18]. In the present study, the performance of the proposed wave indicator was not compared to other measures. For example, it is unclear whether it has additional value in comparison to previously proposed measures such as Bandt and Pompe’s permutation entropy and Wilder’s relative strength index [19].

In the present study, the Bayesian information criterion was used as the basis for estimating the proposed wave indicator. Other information criteria may also fulfill a similar role. For example, the Akaike information criterion can be used to calculate so-called ‘Akaike weights’, which can be interpreted as conditional probabilities for each model [20,21].

An important question concerns the generalizability of the proposed measure. It is largely unclear under which circumstances and in which settings it can be used. In the three case studies included in the present investigation, the findings seemed promising. However, countries may differ regarding various factors, including population demographics, healthcare systems, and government policies. It is necessary to address geographic heterogeneity in future investigations more systematically. It should be noted that using standardized data, i.e., relative instead of raw case counts, does not automatically eliminate geographic heterogeneity [22]. The exploration of heterogeneity regarding transmission dynamics and the general development of infectious disease epidemics with unstandardized or standardized data is likely to be a very important avenue for further research. Accordingly, finding the right balance between local and global measures to mitigate epidemics continues to remain a central issue of policy-making during disease outbreaks.

An interesting characteristic of the proposed measure is that it can also be used to detect phases of exponential decline in new case counts, which was not followed in the present study and did not have received much attention in general yet. Future modeling and empirical studies may explore whether an exponential rather than linear decline may provide valuable information regarding epidemic dynamics.

Given that even central epidemiological concepts lack a consensual definition [23,24], thinking about epidemic waves formally as trends with specific characteristics in time series data may be a fruitful perspective [19]. Although the proposed measure is intended to be a descriptive indicator of epidemic waves, testing its value for prediction might be an interesting avenue of research [18]. In addition, analyzing its agreement with similar measures, such as the average of the effective reproduction number R across a defined period of time [8], could be an informative focus of future studies.

## 5. Conclusions

Early detection of waves during infectious disease epidemics can prevent serious consequences. Approaching this problem from a scientific measurement perspective may offer new insights. In the present study, an epidemic wave indicator was proposed. Even though the presented measure is approximate, relies on simplified assumptions, and needs further evaluation, it may contribute to putting discussions on epidemic waves on a more objective basis.

## Data Availability

Publicly available data from the World Health Organization Coronavirus Disease (COVID-19) Dashboard were analyzed in this study. This data can be found here: https://covid19.who.int.

## Supplementary Materials

The following supporting information can be downloaded at: https://www.mdpi.com/article/10.3390/epidemiologia4030027/s1, Text S1: R Code.
